# CCR5∆32 and SDF1 3′A: Gene Variants, Expression and Influence on Biological Markers for the Clinical Progression to AIDS among HIV-1 Virus Controllers in a Mixed Population of the Amazon Region of Brazil

**DOI:** 10.3390/ijms24054958

**Published:** 2023-03-04

**Authors:** Érica Ribeiro Gomes Lima, Maria Alice Freitas Queiroz, Sandra Souza Lima, Luiz Fernando Almeida Machado, Izaura Maria Vieira Cayres-Vallinoto, Antonio Carlos Rosário Vallinoto, Fernanda Andreza de Pinho Lott Figueiredo, João Farias Guerreiro, Marluísa de Oliveira Guimarães Ishak, Ricardo Ishak

**Affiliations:** 1 Virus Laboratory, Institute of Biological Sciences, Federal University of Pará, Belém 66075-110, Brazil; 2 Human and Medical Genetics Laboratory, Institute of Biological Sciences, Federal University of Pará, Belém 66075-110, Brazil

**Keywords:** HIV-1, CCR5, SDF1, gene variants, viremia controller

## Abstract

CCR5Δ32 and SDF1-3′A polymorphisms were investigated in a cohort of viremia controllers, without the use of therapy, along with their influence on CD4+ T lymphocytes (TLs), CD8+ TLs, and plasma viral load (VL). The samples were analyzed from 32 HIV-1-infected individuals classified as viremia controllers 1 and 2 and viremia non-controllers, from both sexes, mostly heterosexuals, paired with 300 individuals from a control group. CCR5∆32 polymorphism was identified by PCR amplification of a fragment of 189 bp for the wild-type allele and 157 bp for the allele with the ∆32 deletion. SDF1-3′A polymorphism was identified by PCR, followed by enzymatic digestion (restriction fragment length polymorphism) with the Msp I enzyme. The relative quantification of gene expression was performed by real-time PCR. The distribution of allele and genotype frequencies did not show significant differences between the groups. The gene expression of CCR5 and SDF1 was not different between the profiles of AIDS progression. There was no significant correlation between the progression markers (CD4+ TL/CD8+ TL and VL) and the CCR5∆32 polymorphism carrier status. The 3′A allele variant was associated with a marked loss of CD4+ TLs and a higher plasma VL. Neither CCR5∆32 nor SDF1-3′A was associated with viremia control or the controlling phenotype.

## 1. Introduction

Human immunodeficiency virus 1 (HIV-1) infection is an important cause of diseases around the world, and since the discovery of Acquired Infectious Disease Syndrome (AIDS) and its etiological association with HIV-1, the burden inflicted onto the human population is still of a large magnitude. In 2020, there were approximately 37.7 million persons infected with HIV-1 around the world, in which 35.9 million were adults and 1.8 million were children under 14 years of age [1]. In Brazil, 342,459 new cases were reported from 2007 to June 2020, and 30,943 were from the north region of the country [2]. The prevalence of infection in the Amazon region varies according to the group examined; for instance, 0.3% among pregnant adolescents [3], 0.64% in the Marajo Archipelago [4], 2.3% in female sex workers and 3.7% in drug users [5].

The natural history of HIV-1 infection and the progression to AIDS in the absence of therapeutic intervention is variable and can be classified as (i) rapid progression, when AIDS develops in 2–3 years; (ii) typical or intermediate progression, when AIDS develops between 3 and 10 years after infection; and (iii) long-term non-progression, when AIDS develops slowly, such as in elite controllers, rare HIV-1 infected individuals who control viral replication and maintain undetectable viremia, without the intervention of antiretroviral therapy [6,7,8,9,10].

The control mechanisms of the virus and host factors are not yet fully known. Although there is evidence that the viral strains in controllers may be less virulent than the strains of noncontrollers [11], HIV-1 strains isolated from controllers often have replication kinetics similar to those of other in vitro isolates and suggest that viral fitness cannot fully explain the phenomenon [12,13,14]. The virus can be transmitted from a progressor who developed AIDS to a controller, which shows the importance of host factors in the control of infection [15]. The immune response, genetic variations, and gut translocation, which are directly associated with the inflammatory process, may also be involved [16,17,18].

Several host and virus determinants have given us a better understanding of HIV-1 transmission and progression to AIDS. The immune response elicited by CD8+ T lymphocytes (TLs) exerts a major evolutionary pressure on HIV-1, and mutations that allow response escape lead to more rapid progression to AIDS [19,20,21]. Some variants of human leukocyte antigens (HLAs) are associated with a low viral load and slow progression to AIDS (HLA-B*57 and HLA-B*27), while the HLA-B*35 variant is associated with rapid progression [22,23,24,25,26,27].

Genetic variants that explain part of this variation were identified within or near genes encoding the virus entry receptors in cells (such as CCR5 and CXCR4) and molecules involved in the adaptive and innate immune response [28,29]. The CCR5 chemokine receptor is used by HIV-1 as a fundamental coreceptor for HIV-1 fusion and entry into the host cell [30]. At least 16 gene variants in the CCR5 gene generate changes in the encoded amino acid. Among them is the deletion of 32 bp called CCR5Δ32, which has been the most investigated in association with several pathologies [31,32,33,34,35,36]. The homozygous CCR5Δ32 mutation reduces the risk of HIV-1 acquisition, while the effect of the heterozygous mutation appears to be null [37,38] or promotes a slower progression to AIDS [39].

Chemokine CXCL12, also known as stroma-derived factor-1 (SDF1), is the ligand of chemokine receptor CXCR4 [40]. The change from guanine (G) to adenine (A) at position 801 (3′ untranslated region, 3′ UTR) counting from the ATG start position is a gene variant designated SDF1-3-prime UTR-801G-A (abbreviated SDF1-3′A) or rs1801157 [41]. The A allele is associated with increased levels of SDF1 mRNA and enhanced mRNA stability, which has a half-life twice as long as the 3′G variant [42]. The A allele results in an increased SDF1 concentration and is associated with protection against HIV-1 infection, especially against syncytium-inducing (SI) variants of HIV-1 at the CXCR4 receptor level [41,43].

The identification of markers that induce a more efficient response to HIV-1 infection and control of the infection is a challenge, including for the development of a prophylactic or therapeutic vaccine. The evaluation of the gene variants in HIV-1 receptor proteins expressed on the cell surface and their natural ligands may improve our understanding of spontaneous infection control. The objective of the present study was to evaluate the inherent contribution of the host to the control of viral replication in terms of the CCR5Δ32 and SDF1-3′A gene variants and the gene expression of CCR5 and SDF1 in the different progression profiles of HIV-1 infection.

## 2. Results

### 2.1. Epidemiological Variables, Gene Variants and Gene Expression

The epidemiological characteristics of the VC1, VC2, and NC groups were not associated with sex ratio, age, education, sexual behavior, or other risk behavior (Table 1).

The frequency distribution of the genotypes was consistent with the Hardy–Weinberg equilibrium in the VC1, VC2, NC and control groups for the genetic variations CCR5∆32 and SDF1-3′A. 

The wild-type genotype of CCR5Δ32 was the most frequent among the groups. The Δ32 allele variant was not found in either VC1 nor VC2, but was twice as frequent among the NCs (7.14%) than among the control group (3.5%). Only one individual homozygous for the Δ32 allele (0.33%) was found in the control group. There was no significant difference in the distribution of genotypes or alleles between the investigated groups (Table 2).

For the SDF1-3′A gene variant, the frequency of variant genotypes (GA and AA) was higher in the NC group, compared to the VC1, VC2 and control groups, with a *p* value close to the level of statistical significance (*p* = 0.0779). A higher frequency of the variant allele (A) was also observed in the NC group, but without statistical significance (Table 2).

**Figure 1:**
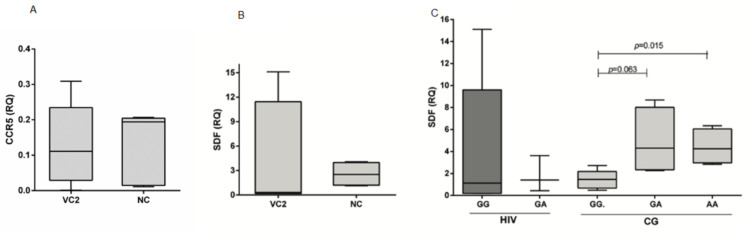
Levels of CCR5 mRNA (**A**) between viremia 2 controllers (VC2) and non-controllers (NC); (**B**) levels of SDF1 mRNA between VC2 and NC; and (**C**) according to the genotype of the SDF1-3′A variant (rs1801157) in HIV-1 infected individuals and in the control group (CG). RQ: relative quantification.

Some samples were not of a good enough quality to be processed adequately (degraded RNA or low concentration of RNA) and, consequently, they were not amplified for the targets (CCR5 and SDF) or for the endogenous control (GAPDH). The quantification of CCR5 mRNA was performed in 10 samples from HIV-1 infected individuals (five VC2 and five NC) and 14 individuals from the control group. CCR5 mRNA levels were higher among NC subjects (median = 0.194) than among VC2 subjects (median = 0.111), but not statistically significant (Figure 1A). A comparison of the gene expression level in relation to the genotypes for the CCR5∆32 gene variant of HIV-1 infected individuals was not possible due to the small sample size.

Eight infected samples (four from the VC2 group and four from the NC group) and thirteen control samples were submitted for SDF1 gene expression analysis. The highest expression was in the NC group (median: 2.522), and the lowest in the VC2 group (median: 0.3165), but without statistical significance (Figure 1B). Individuals homozygous for wild-type SDF1-3′A (GG) had lower SDF1 expression. A comparison of the expression levels of the SDF1-3′A genotypes among those infected with HIV-1 (Figure 1C), showed no significant difference. In the control group, the carriers of wild-type genotypes (GG; median: 1.649) had significantly lower levels of expression compared to individuals with heterozygous variants (AG; median: 4.252; *p* = 0.063) and with homozygous variant genotypes (AA; median: 4.3095; *p* = 0.015).

### 2.2. Associations between CD4+ T and CD8+ T Lymphocytes and HIV-1 Plasma Viral Load with Infection Progression and Genetic Variants 

**Figure 2:**
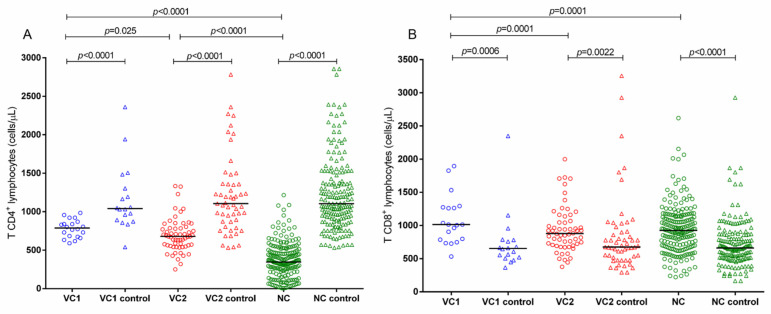
Comparison of (**A**) CD4+ TL counts and (**B**) CD8+ TL counts between non-infected and HIV-1 infected persons, according to the control progression of infection.

The mean annual variations in the percentages of CD4+ T and CD8+ TLs between VC1, VC2 and NC for HIV-1 infection and their respective controls are shown in Figure 2. VC1 and VC2 had significantly higher CD4+ TL counts (medians = 757 and 672, respectively) than NC (median: 357) (Figure 2A). In contrast, the CD4+ TL levels of the VC1 and VC2 groups were significantly lower than the matched controls (median for VC1 control = 1041, for VC2 = 1106 and for NC = 1105; Figure 2A).

CD8+ TL was significantly more abundant in the VC1 group (median = 1025) than in the VC2 (median = 846) and NC (median = 912) groups (Figure 2B). All three groups had significantly higher values than the uninfected controls (median for VC1 control = 653, for VC2 = 674 and for NC = 660; Figure 2B).

The individual evolution of the CD4+ T and CD8+ LT biomarkers and the HIV-1 plasma viral load is shown for each individual in the Supplementary Figure S1 (VC1), Supplementary Figure S2 (VC2) and Supplementary Figure S3 (NC), starting from the first available measurement.

**Figure 3:**
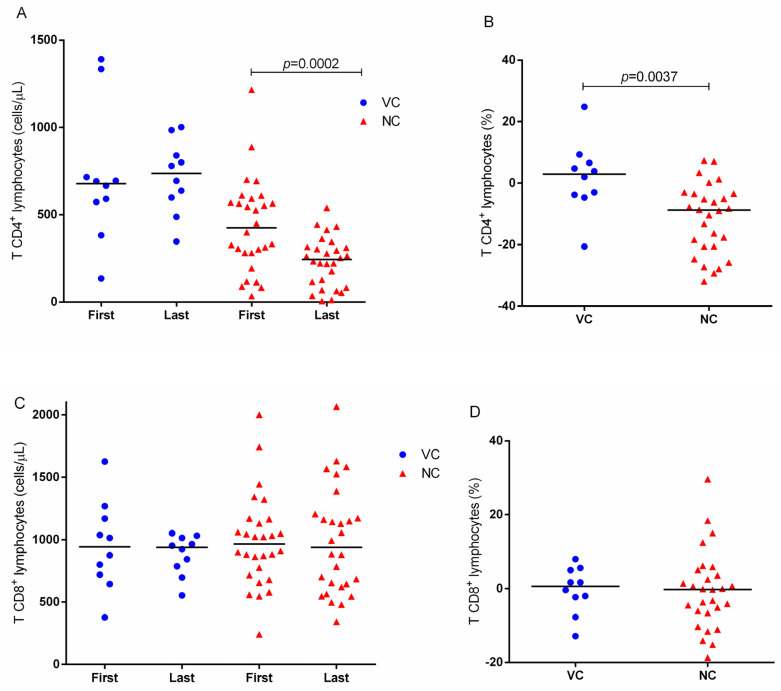
Longitudinal evolution of CD4+ TL and CD8+ TL counts in viremia controllers (VC) and non-controllers (NC). (**A**) Quantifications of CD4+ TL, absolute values. (**B**) Mean annual variation in the CD4+ TL count calculated by the compound annual growth rate (CAGR). (**C**) Quantification of CD8+ TL, absolute values. (**D**) Mean annual variation in the CD8+ TL count calculated by CAGR.

The analysis of the mean annual variation of the CD4+ and CD8+ TL counts was based on the CAGR, which describes the annual growth rate over a given time. VC1 and VC2 counts were merged into a single group of controllers in order to improve the analysis. Between the first and last lymphocyte count, there was a slight increase (without statistical significance) in the CD4+ TL level in the viremia controllers and a significant decrease in the NC group (Figure 3A). The annual variation in the CD4+ TL count was also significantly different between these two groups, with greater cell loss in the NC group (Figure 3B). There was no difference in the evolution of the CD8+ T cell count or in the annual growth rate between the controllers and NCs (Figure 3C,D).

**Figure 4:**
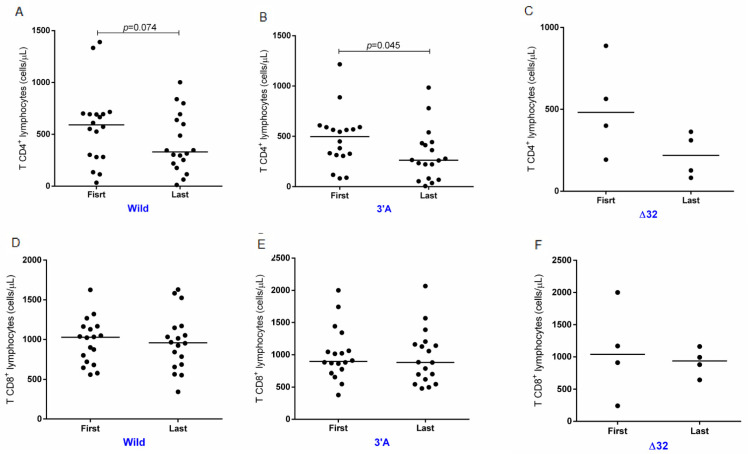
Longitudinal evolution of LT CD4+ and LT CD8+ counts in individuals with HIV-1 carrying the wild-type genotype and variants for SDF1-3′A and CCR5Δ32. CD4+ LT quantifications of carriers (**A**) of the wild-type genotypes, (**B**) of the SDF1-3′A variant and (**C**) CCR5Δ32. CD8+ LT quantifications of carriers (**D**) of the wild-type genotypes, (**E**) of the SDF’-3′A variant and (**F**) CCR5Δ32.

CAGR was also calculated for CD4+ and CD8+ TL according to the studied gene variant to search for associations between disease progression and the genetic profile of the infected individual. There was a marked decrease in the CD4+ TL count in all the groups evaluated, but this decrease was only statistically significant in individuals with the presence of the 3′A allele variant (Figure 4A–C). The evolution of the CD8+ TL count according to the presence of gene variants was stable in all groups, with no significant differences (Figure 4D–F).

**Figure 5:**
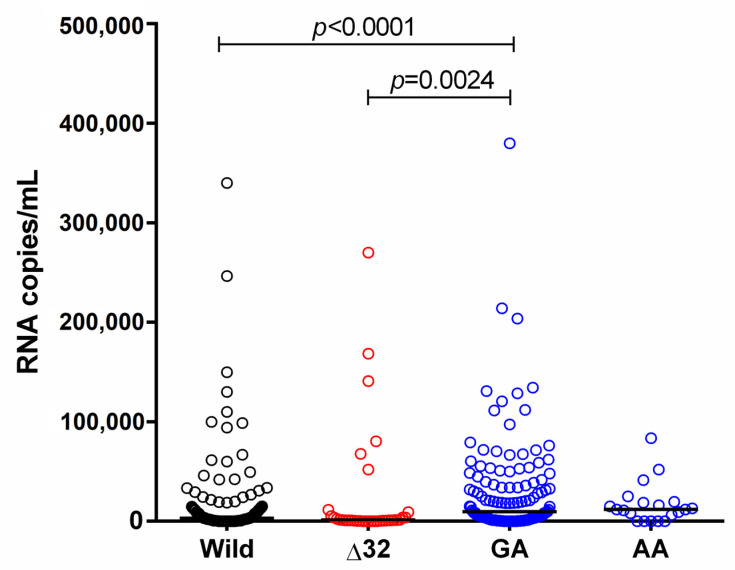
Plasma viral load of HIV-1 in the groups with the different genotypes of the CCR5∆32 and SDF1-3′A gene variants.

The HIV-1 plasma VL was compared between the different genotypes of the studied variants, and it was significantly higher among the heterozygotes for the SDF1-3′A variant (Figure 5). 

When the VL was segmented into quintiles (from undetectable to values greater than 100,000 copies/mL, log10-adjusted), the relative representations of the three genotypes were statistically significant according to the different quintiles. The undetectable viral load quintile (<50 copies/mL) was over-represented by individuals with the wild-type genotype. Counts of 50–1000 copies/mL were significantly over-represented with individuals with the ∆32 allele, while the counts of 10,000–100,000 copies/mL were significantly over-represented by individuals with the 3′A allele (Table 3).

## 3. Discussion

This study is the first to investigate the occurrence of CCR5∆32 and SDF1-3′A gene variant genotypes in HIV-1 infection controllers in north Brazil. The importance of studying this population group is because the state of Para is located in the north of Brazil and presents peculiarities when compared to the other geographical areas of the country. The genetic background of the peoples inhabiting the Amazon region of Brazil, resulting from the interethnic mix of the indigenous populations already residing in the area, the white European colonizers (mainly from Portugal), and the Black African slaves brought in for more than 350 years, since the 17th century. This genetic mix occurred all over the country, with a different component from North to South ranging from small contributions to very large ones according to the region involved. For instance, in northern areas, the original Indigenous contribution is higher than those observed in southern regions, where Europeans are predominant.

The influence of CCR5Δ32 and SDF1-3′A gene variants was investigated in a cohort of VC and NC matched with a control group. Viral controllers are fewer than 1% of HIV-1 infected persons and occur in the absence of antiretroviral therapy [6,7,8,44]. Cohorts referred to as elite suppressors, HIV-1 controllers, elite controllers, and natural controllers have been described [7,45,46], and apparently represent the natural adaptation of the virus to the host. In contrast, there is little information on HIV-1 control in the northern region of Brazil [47,48].

The classification of viral replication controllers is still pending consensus [7,45,49,50]. The criteria used in the present study were previously published [47,51] and included the CD4+ TL count (minimum of 500 cells/mm3, in 90% of the measurements), undetectable plasma viral load, and at least 6 years of observation (reaching up to 11 years).

There are several descriptions of viral controllers, and they are similar to what was found in the present study regarding the frequency of VC1 (0.6%), infection for 15.5 years (median), maintaining a heterosexual relationship, CD4+ TL count of 757 cells/µL (median), and a median time of viral replication control greater than 8 years [52,53,54]. The demographic information of the investigated group was not different from that of the general population infected by HIV-1 in Brazil. There has been no reported predominance of sex or modes of HIV-1 infection, nor demographic or ethnic differences, in controllers, with the exception of a cohort of people of African descent in the USA [52]. The epidemiological variables are similar between the VC1, VC2, and NC progression profiles [47,55,56].

The participants of the present study are part of a group with genetic markers of Amerindians, Europeans, and Africans [57,58,59,60], and the frequency found (7.14%) of the ∆32 allele variant in HIV-1 carriers and among noninfected individuals (3.5%) is compatible with that found in the north region of Brazil [61,62,63,64] and among all HIV-1 infected individuals [65,66,67]. The frequencies of the ∆32 and 3′A alleles are consistent with the observation that they vary between ethnic groups [68,69,70,71], and the group examined was highly mixed [57].

CCR5 is a coreceptor exploited predominantly by M-tropic strains of HIV-1, and its expression is associated with the progression to AIDS [72,73,74]. The CCR5Δ32 variant prevents binding to HIV-1, but does not affect the level of mRNA expression [37,68], although the presence of the Δ32 allele and other gene variants in the promoter region of the CCR5 gene [75,76] is associated with higher levels of CCR5 gene expression among HIV-1 replication controllers [77]. The Δ32 variant was not found among VC1 or VC2 members, and the allele and genotype frequencies did not differ significantly among cohorts in Brazil and the USA [46,54,78]. The absence of an association between gene variants and infection controllers might be masked by the occurrence of two rare events (the low number of controllers and the low frequency of the Δ32 allele). There was no difference in the levels of CCR5 gene expression, either between people with the presence vs. the absence of the Δ32 allele or in comparison with the control group (which had the only homozygous genotype).

The Δ32 allele was present in heterozygosity, but did not influenced the evolution of the CD4+ TL or CD8+ TL counts or when compared to other genetic profiles (wild-type for Δ32 and 3′A and presence of the 3′A allele). The absence of association of the variant with the levels of CD4+ TL differs from the suggestion that the heterozygous Δ32 allele is associated with a slower progression to AIDS [36,37,76,79,80,81,82,83]. Protection is gradually lost when viral tropism ceases to be M-tropic, begins using the CXCR4 receptor, and becomes preferentially T-tropic and is clearly associated with a rapid decline in CD4+ TL [84,85,86]. Of the four individuals, two [Supplementary Figure S3, NC II and NC XVII] may have been re-infected by a dual-tropic strain, or they may have had a change in viral tropism, losing the protective effect of the CCR5∆32 variant; both had decreased CD4+ TL despite the low rate of viral replication. NC II was diagnosed in 1988 and was followed up for 9 years, showing a progressive loss of CD4+ TL and mild control of the viral load (<5000 copies/mL, consistently). Patient XVII was followed for 9 years, with CD4+ TL levels consistently below 500 cells/µL and surprising viral load control (<1000 copies/mL consistently). Due to the nature of the study, it was not possible to define the viral phenotype to test the suggested hypothesis.

The 3′A allele variant was found, in homozygosity and heterozygosity, among VC1, VC2, NC, and control participants, with a homogeneous distribution of the allele and genotype forms, which does not indicate an association between the variant and susceptibility to certain infection or progression phenotypes. Similar frequencies of alleles and variants between typical controllers and progressors have been previously reported [77,82,87,88]. The 3′A allele variant has been correlated with an elevated SDF1 mRNA expression and increased SDF1 chemokine concentration in healthy individuals [42], which was corroborated by the significantly higher expression in individuals with the GA and AA genotypes in comparison with those with the GG genotype in the control group.

The levels of SDF1 are considered to be high among HIV-1 viral replication controllers [49,77], which was not found among VC1 and VC2. The small sample size and the additional segregation according to genotype may have reduced the chance of demonstrating an effect on the SDF1 level. The recommendation of immediate treatment [89] after diagnosis of HIV-1 infection (“test and treat”), makes the evaluation of a rare event even more difficult to study.

The influence of the SDF1-3′A variant on HIV-1 infection is still controversial. The 3′A allele has shown a positive influence in delaying the progression to AIDS [41,90,91], and with higher rates of CD4+ TL decline and progression to AIDS [92,93,94,95,96]. In our cohort, the mean annual variation in the CD4+ TL or CD8+ TL count was not different between the groups considering or the allele variants, but there was a significant decline in CD4+ TL among the carriers of the 3′A allele variant and not to those with the ∆32 allele variant or the wild-type allele. The plasma VL was also significantly higher among carriers of the 3′A variant, suggesting an accelerated progression to AIDS.

SDF1 is the only natural ligand of CXCR4, and the change in viral tropism may occur by altering the expression of SDF1 induced by the 3′A allele. The follow-up of a cohort for 10 years suggested the involvement of the 3′A allele with a higher probability of presenting X4/IS strains and faster progression to AIDS [94]. In contrast, this association was not seen in an in vitro tropism evaluation between the SDF1-3′A genotypes and the prevalence of primary IS/NIS isolates [92,93]. The levels of SDF1 expression are divergent and may delay the progression to AIDS [41,97,98,99] or favor the progression to AIDS [93,94,100]. The 3′A allele may be associated with higher [97,100] or lower SDF1 expression [87] or in association between genotypes and expression levels [101]. Similarly, a positive or negative correlation between SDF1 protein levels and CXCR4 expression in CD4+ TL has been observed, which varied according to the stage of infection [100].

The different definitions of viremia controllers is the most probable cause for the great divergence of the published results. The maintenance time for the minimum conditions of infection control should be included and followed throughout the observation period. The inclusion of persons who spend months or a few years with a sustained CD4+ TL count and low or no viral load should not be acceptable. Other variables need to be characterized, such as the natural ligands of CCR5 RANTES and the macrophage inflammatory protein 1α (MIP-1 α) [10,102]; immune response genes—the human leukocyte antigen (HLA) and NK cell immunoglobulin-like receptor [50,103,104], β-defensin [105], and Toll-like receptors [106]; and the profile of expressed cytokines [47,49,55], which play a suppressive role in HIV-1 replication and affect the natural history of HIV-1 infection. The variations in the CCR5 and SDF genes may not be sufficient to control the viral load and restore the CD4+ TL repertoire.

No VL blips were observed among the VC1, but they were observed among the VC2, although they were controlled without the use of ART, even when the VL reached up to 10,000 copies. The time interval for measuring the HIV-1 plasma viral load sometimes reached six months apart. It is possible that viral blips could have been missed among the viral controllers. However, if that was the case, recovery would occur efficiently without the use of ART. Viral persistence in the lymph nodes was not sufficient to act as a virus repository [55,107,108], as observed among controllers.

The ∆32 allele and the 3′A allele were found more frequently among individuals in different quintiles of VL (1000 copies/mL and up to 100,000 copies/mL). Persistent activation of the immune system to HIV-1 infection leads to high levels of CD8+ TL activation, and a progressive decline in CD4+ TL [109,110]. Even among viremia controllers, in the absence of ART, viremic episodes induce chronic immune activation and inflammation and decrease CD4+ TLs [7,46,78]. The heterozygosity (GA) for the SDF1-3′A variant showed a possible influence on the plasma VL of HIV-1, and the distribution of VL values was lower among individuals with the wild-type genotype, which indicates that they provide better spontaneous control of the VL than those with an allele variant.

CD4+ TL decreased significantly among the NC, which is a common occurrence among non-controllers [110]. However, the counts and the evolution of the counts, measured by CAGR, were consistently high among the viremia controllers, even if they were below the median found in the control group. The groups of viremia controllers are usually heterogeneous, and there are conflicting results, with some controllers experiencing an initial increase in CD4+ TL followed by temporary stability and others showing a gradual decrease [111], with their CD4+ TL counts remaining stable and without evidence of immunological activation [112] or exhibiting levels of CD4+ TL and CD8+ TL activation and significant loss of CD4+ TL [110,113].

The depletion of CD4+ TL is usually accompanied by the inversion of the relationship with CD8+ TL [114]. In our cohort, the CD8+ TL counts and the evolution of the counts, measured by CAGR, in the controllers and non-controllers were continuously stimulated and were significantly higher than those in the noninfected controls. CD8+ TL play a role in the pathogenesis of HIV-1, but it is thought that there is an increase in the strength of the immune response [115,116] to change the outcome of the infection by suppressing viral multiplication and decreasing the viral load [117,118], by maintaining adequate levels of IFN-γ, which acts on the innate response, and by means of cytotoxic CD8+ TL action [119,120].

The present study shows an association between the 3′A allele variant and the decline in CD4+ TL, probably due to an increased expression of SDF1 mRNA during the more advanced stages of HIV-1 infection, but the presence of the CCR5∆32 and SDF1-3′A gene variants were not associated with a better response against viral multiplication or control by the host.

It is a common observation that the occurrence of persons considered as replication controllers is a very rare event and, consequently, this makes it difficult to conduct appropriate statistical tests. Conversely, the use of methods that mimic epidemiological studies applied to rare events, as the small numbers of viremia controllers (VC1 and VC2), the amplification of numbers by counting all variables as multiple persons (similarly to the definition of “person/years” in rare epidemiological events) and the use of statistical methods such as CAGR, which is commonly used in economics to evaluate trends, are innovative approaches to improve the understanding of the influence of the polymorphism.

Although lymphocyte functionality was not measured, the relative and absolute counts of CD4+ TL and CD8+ TL should continue to be relevant variables for the maintenance and monitoring of HIV-1 carriers, be they controllers or non-controllers.

## 4. Materials and Methods

### 4.1. Type of Study and Selection Criteria

The present study is a retrospective cohort of HIV-1 infected persons attending the AIDS State Reference Unit continuously and on a regular basis (at least 6 years) from 2007-2015, according to their medical records, which served as the basis for a prospective clinical study of virological progression, host response and to define host genetic variants. HIV-1 viremia controllers are extremely rare, and their detection is a difficult task during a routine clinical observation by a physician, as it takes some time to define the chance of viral replication control. Consequently, they could not be selected at random (or other types of recruitment) or define the sample calculation. The official decision of the Brazilian Ministry of Health for testing everyone as soon as the laboratory diagnostic is defined (“test and treat”) made it an even rarer opportunity to understand the pathogenesis of HIV-1 a bit further with this selective group of persons.

The criteria for defining the groups involved were previously published [47]. Viremia controllers 1 and 2 (VC1 and VC2) and viremia non-controllers (NC) were defined according to their number of years postdiagnosis (at least 6 years), CD4+ and CD8+ TL counts, and plasma viral load (VL), with measurements at least twice a year, in the absence of specific HIV-1 therapy.

Table 4 lists the main characteristics of the three groups (VC1 = 2; VC2 = 8; NC = 28). They were all treatment naïve, and the initiation of antiretroviral therapy (ART) at any time resulted in exclusion from the study. Individuals older than 18 years of age of both sexes residing in the state of Pará, Brazil, seen at the Reference Unit for Special Infectious and Parasitic Diseases (URE-DIPE), who are part of the monitoring of the National Network of CD4+ TL count and plasma VL, were included in the study. The samples were part of the collection of biological materials of the Laboratory of Virology (LabVir), Institute of Biological Sciences, Federal University of Pará (ICB/UFPA). A control group of 300 non-infected individuals was recruited at random in order to compare the host genetic variants between the control individuals and the infected patients. The control samples were obtained from regular blood donors of the Fundação HEMOPA and were matched (seven to nine uninfected to each infected person) by sex and age (range of five years up or down).

The project was approved by the Ethics Committee of the Tropical Medicine Center of the Universidade Federal do Para (#275.456), and informed consent was obtained from all subjects involved in the study.

### 4.2. Data Collection

The behavioral and risk factors for HIV-1 infection were assessed based on data collected from the LabVir-ICB/UFPA database obtained in interviews with the participants. The data were collected from 2007 to 2015 for the quantification of CD4+ TL/CD8+ TLs and VL from the database of the Laboratory Examination Control System of the National Network of CD4+/CD8+ Lymphocyte Count and Viral Load (SISCEL), of the Brazilian Ministry of Health. Information on the use of ART was obtained by consulting the medical records of URE-DIPE and the Logistic Control System of Antiretroviral Medicines (SICLOM), also from the Brazilian Ministry of Health, which records and manages the dispensing of antiretroviral drugs to patients of Brazil’s national health system network.

### 4.3. Quantification of the Plasma Viral Load of HIV-1 and CD4+/CD8+ TLs

CD4+ TL and CD8+ TLs were quantified by flow cytometry (BD FACSCaliburTM, Becton & Dickinson, Franklin Lakes, NJ, USA) with the FACSCountTM Reagents monitoring kit, following the protocol recommended by the manufacturer (Becton & Dickinson, Franklin Lakes, NJ, USA). The viral load was quantified by real-time PCR using the Sample Purific CV HIV-1 extraction kit (Abbott, Chicago, IL, USA) and the HIV-1 viral load amplification kit (Abbott, Chicago, IL, USA). The units used were copies/mL and log10. The determinations followed the standard established by the National Network for the Determination of CD4+ and CD8+ T cells and Viral Load of the Department of HIV/AIDS and Viral Hepatitis of the Brazilian Ministry of Health. A statistical evaluation of the plasma levels of CD4+ and CD8+ TL was performed on the median values of each group.

### 4.4. Identification of CCR5Δ32 and SDF1-3′A Polymorphisms

The DNA was extracted from peripheral-blood leukocytes by the phenol–chloroform method [121]. The genotypes of CCR5Δ32 and SDF1-3′A were previously defined [41,46]. The CCR5Δ32 gene variants was identified by conventional PCR in the amplification of 189 bp for the wild-type allele and 157 bp for the Δ32 allele of the CCR5 gene, as previously described [80]. For the SDF1-3′A gene variant, conventional PCR followed by restriction fragment length polymorphism analysis was used, in which the amplified products were incubated with restriction endonucleases, visualized, and compared on fragment sizes, as described previously [41]. The PCR-RFLP protocol included the Sanger sequencing for the verification of the efficiency and reproducibility of the method in the detection of wild and mutant alleles of both genes.

### 4.5. RNA Extraction

RNA was extracted from peripheral-blood leukocytes with the Total RNA Purification Kit (Norgen, Biotek Corporation^®^, Thorold, ON, Canada) following the manufacturer’s instructions. The RNA concentration (ng/µL) was obtained by analysis on the QubitTM Quantitation Platform fluorimeter (Invitrogen^®^, Waltham, MA, USA). The concentration of each total RNA sample was adjusted to 60 ng/µL for cDNA synthesis.

### 4.6. Reverse Transcription for Complementary DNA (cDNA) Synthesis

The extracted RNA was converted into cDNA using the High Capacity cDNA Reverse Transcription^®^ with RNase Inhibitor kit (Applied Biosystems, Foster City, CA, USA). For the reaction, a mix was prepared with a final volume of 20.0 µL containing 2 µL of 10× RT Buffer, 0.8 µL of 25× dNTP Mix (100 nM), 2 µL of random primer, 1 µL of MultiScribeTM Reverse Transcriptase, 1 µL of RNaseOUTTM, and 3.2 µL of ultrapure water, provided by the kit, plus 10.0 µL of extracted RNA. The mixture was run in a Mastercycler Personal thermocycler (Eppendorf, Hamburg, Germany) at 25 °C for 10 min, at 37 °C for 120 min, and at 85 °C for 5 min.

### 4.7. Gene Expression

The qPCRs were standardized with the cDNA and probes (endogenous genes and target genes) to calculate the efficiency of the amplification reactions by testing different cDNA concentrations (undiluted, 1:2, 1:4, 1:8, and 1:16) in triplicate plate wells (the same cDNA in different dilutions and with different probes) for the construction of the efficiency curve and validation of the 2-ΔΔCT analytical method. We assumed that all assays had an efficiency of 100% (±10) [122]. 

The reactions were performed in the StepOnePLUS™ Real-Time PCR System (Applied Biosystems, Foster City, CA, USA) according to the manufacturer’s protocol. The TaqMan^®^ Gene Expression Assay (Applied Biosystems, Foster City, CA, USA) used primers and probes specific to each target gene (CCR5: Hs00152917_m1; SDF1: Hs00171022_m1) and the endogenous control GAPDH (HS02758991_g1). Each reaction had 15 μL of 2× TaqMan^®^ Universal PCR Master Mix, 1.5 μL of 20× TaqMan Gene Expression Assays, 3 μL of cDNA, and 10.5 μL of RNase-free water. The thermocycling conditions were 2 min at 50 °C, 10 min at 95 °C, and 40 cycles of 15 s at 95 °C and 1 min at 60 °C.

### 4.8. Statistical Analysis

The epidemiological and behavioral characteristics are described using descriptive statistics; the categorical variables are presented as frequencies and percentages, and the numerical variables are presented as median and quartile spread or mean and standard deviation. The numerical variables were evaluated for the normality and homogeneity of variances by the Kolmogorov–Smirnov and Levene tests, respectively.

The G test was used to compare the epidemiological and behavioral characteristics and the distribution of genotype and allele frequencies between the groups. The evaluation of the levels of gene expression in relation to the studied groups and the genotypes was performed by the Mann–Whitney and ANOVA or Kruskal–Wallis tests, respectively.

In order to approach the problem of analyzing the information dealing with a restricted number of persons or rare events (such as the viremia controllers), a similar definition of “number of persons/years”, a common definition in epidemiological studies, was adopted. All the available measures of T CD4+, T CD8+ lymphocyte counts and plasma viral load were used and considered to be from a single individual measure. The use of the compound annual growth rate (CAGR) for the longitudinal analysis (a method which is used in economics) is also a commonly accepted statistical approach for this particular situation [55]. With this practice, the numbers are brought to a reasonable figure, and sufficient to allow a proper statistical evaluation.

The Kruskal–Wallis and Mann–Whitney tests, respectively, were applied to compare the levels of the CD4+ and CD8+ TLs between the groups and the levels of each group with their respective controls. An analysis was performed on the mean annual variations of the percentages of the CD4+ T and CD8+ TLs, which were calculated by their CAGR [55]. The results of this variation were compared between the VC and NC groups and according to the genetic profile by using the Mann–Whitney test. The paired Wilcoxon test was used to evaluate between the first and the last CD4+ and CD8+ TL counts.

The Mann–Whitney test and the G test were performed to find any association between the genetic profile and the plasma VL. The tests were run in BioEstat 5.3 software and GraphPad Prism software. Associations with *p* < 0.05 were considered significant.

## 5. Conclusions

Knowledge about the characteristics of HIV-1 viremia controllers, including their genetic background, is useful in order to learn about the pathogenesis of the virus. The recent adoption of a new protocol for immediate treatment at diagnosis will further reduce the chances of finding controllers and forming new cohorts that allow long periods of observation without the use of therapy. The influence of genetic markers, particularly the CCR5∆32 and SDF1-3′A polymorphisms, on the relationship between controllers and non-controllers of HIV-1 viremia remains unclear.

## Figures and Tables

**Table 1:** Epidemiological and behavioral characteristics of HIV-1-infected individuals.

	VC1 (*n* = 2)		VC2 (*n* = 8)		NC. (*n* = 28)		*p*
**Sex**							
Male	1	(50%)	3	(37.5%)	16	(57%)	0.6571
Female	1	(50%)	5	(62.6%)	12	(43%)	
**Age (median; Q1 and Q3)**	39.5	(38–41)	31	(28–36)	35	(28–39)	0.5695
**Number of school years**							
Illiterate	0	(0)	1	(12.5%)	0	(0)	0.9098
Primary Education	1	(50%)	3	(37.5%)	15	(53%)	
Secondary Education	1	(50%)	3	(37.5%)	10	(36%)	
Higher Education	0	(0)	1	(12.5%)	3	(11%)	
**Sexual behavior**							
Heterosexual	2	(100%)	6	(75%)	18	(64%)	0.5251
Homosexual man	0	(0)	0	(0)	5	(18%)	
Bisexual	0	(0)	2	(25%)	5	(18%)	
**Other risk behavior**							
IVDU	0	(0)	0	(0)	2	(7%)	0.7305
NIDU	1	(50%)	5	(62%)	18	(64%)	0.9340
Sexual intercourse without a condom	1	(50%)	2	(25%)	13	(46%)	0.5759
Anal sex	0	(0)	4	(50%)	17	(60%)	0.2080
Sex with sex worker	1	(50%)	0	(0)	5	(18%)	0.2178
HIV^+^ partner	2	(100%)	3	(37%)	14	(50%)	0.2430

IVDU: intravenous drug user; NIDU: non-intravenous drug user; G test.

**Table 2:** Distributions of genotype and allele frequencies at the CCR5Δ32 and SDF1-3′A genetic variants (rs1801157).

Genotype and Allele Profile	VC1 (*n* = 2) *n* (%)	VC2 (*n* = 8) *n* (%)	NC (*n* = 28) *n* (%)	Control Group (*n* = 300) *n* (%)	*p*
***CCR5*Δ32**					
*CCR5*/CCR5	2 (100)	8 (100)	24 (85.7)	280 (93.33)	0.7223
*CCR5*/Δ32	0	0	4 (14.3)	19 (6.34)	
Δ32/Δ32	0	0	0	1 (0.33)	
* *CCR5*	4 (100)	16 (100)	52 (92.86)	579 (96.5)	0.3836
* Δ32	0	0	4 (7.14)	21 (3.5)	
***SDF1*-3′A**					
G/G	1 (50)	7 (87.5)	12 (42.86)	180 (60.0)	0.0779
G/A	0	1 (12.5)	15 (53.57)	107 (35.67)	
A/A	1 (50)	0	1 (3.57)	13 (4.33)	
* G	2 (50)	15 (93.75)	39 (69.64)	467 (77.83)	0.0910
* A	2 (50)	1 (6.25)	17 (30.36)	133 (22.17)	

* G test.

**Table 3:** Plasma viral load of HIV-1 according to the presence of variant alleles of CCR5 and SDF1 3′A.

Alleles	Wild-Type (*n* = 18)		∆32 (*n* = 4)		3′A (*n* = 18)		*p*
**Total quantifications**	**153**	**-**	35	**-**	142	-	
**Viral Load**	***n***	**%**	***n***	**%**	***n***	%	<0.0001 *
<50	18	11.8	2	5.7	6	4.2	
50|—1000	33	21.6	11	31.4	16	11.3	
1000|—10,000	59	38.5	15	42.	45	31.7	
10,000|—100,000	36	23.5	4	11.4	68	47.9	
≥100,000	7	4.6	3	8.6	7	4.9	

* G test.

**Table 4:** Distribution of the groups examined, according to sex and age, and criteria for defining the viremia controller and non-controller groups.

Groups According to Disease Progression	Males Age (Range)	Females Age (Range)	Length of Infection (Years)	CD4 + T Lymphocytes	HIV-1 Viral Load	Remarks
**VC1** VIREMIA CONTROLLERS1	1 (52)	1 (45)	>6	>500 cells/mm^3^	<50 copies/mL	No episodes of viral load increase; CD4^+^ T > 500 cells/mm^3^ in 90% of measurements; stable for more than 6 years; no ART intervention
**VC2** VIREMIA CONTROLLERS 2	3 (34–47)	5 (35–53)	>6	>500 cells/mm^3^	≤log_10_4 (≤10,000 copies/mL)	Episodes of increased HIV-1 viral load; decrease in CD4^+^ TL in ~40% of counts; natural remission to regular levels without ART intervention
**NC** NON-VIREMIA CONTROLLERS	16 (28–68)	12 (24–68)	>6	<500 cells/mm^3^	>log_10_4 (>10,000 copies/mL)	

## Data Availability

The data analyzed in this study are included within the paper.

## Supplementary Materials

The following supporting information can be downloaded at: https://www.mdpi.com/article/10.3390/ijms24054958/s1.
