# Supplementary material for: CCR5∆32 and SDF1 3′A: Gene Variants, Expression and Influence on Biological Markers for the Clinical Progression to AIDS among HIV-1 Virus Controllers in a Mixed Population of the Amazon Region of Brazil
Source: Int J Mol Sci. 2023 Mar 4;24(5):4958. doi: 10.3390/ijms24054958 (PMC10003039; doi:10.3390/ijms24054958)
Supplement: Supplementary file 1 [file ijms-24-04958-s001.zip › ijms-2164915-supplementary-done.pdf]

International Journal of *Molecular Sciences*

MDPI

Supplementary Material

CCR5Δ32 and SDF1 3′A: Gene Variants, Expression and Influence on Biological Markers for the Clinical Progression to AIDS Among HIV-1 Virus Controllers in a Mixed Population of the Amazon Region of Brazil

Érica Ribeiro Gomes Lima[1], Maria Alice Freitas Queiroz[1]*, Sandra Souza Lima[1], Luiz Fernando Almeida Machado[1], Izaura Maria V Cayres-Vallinoto[1], Antonio Carlos Rosário Vallinoto[1], Fernanda Andreza de Pinho Lott Figueiredo[2], João Farias Guerreiro[2], Marluísa de Oliveira Guimarães Ishak[1] and Ricardo Ishak[1]

[1] Virus Laboratory, Institute of Biological Sciences, Federal University of Pará, Belém, Brazil

[2] Human and Medical Genetics Laboratory, Institute of Biological Sciences, Federal University of Pará, Belém, Brazil

* **Correspondence:** Maria Alice Freitas Queiroz; alicefarma@hotmail.com

Supplementary Figures

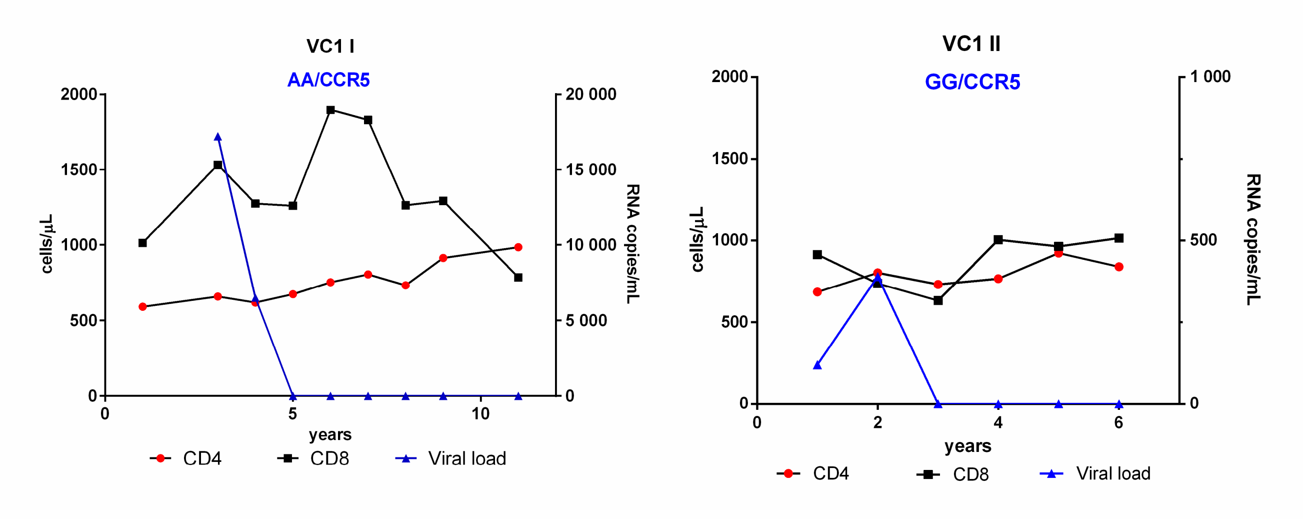

Supplementary Figure S1. Evolution of CD4+ TL and CD8+ TL counts and HIV-1 plasma viral load from the year of diagnosis or the first available quantification among viremia controller 1 (VC1). AA: homozygous mutant for the *SDF1*-3′A variant. GG: wild homozygous for the *SDF1*-3′A.

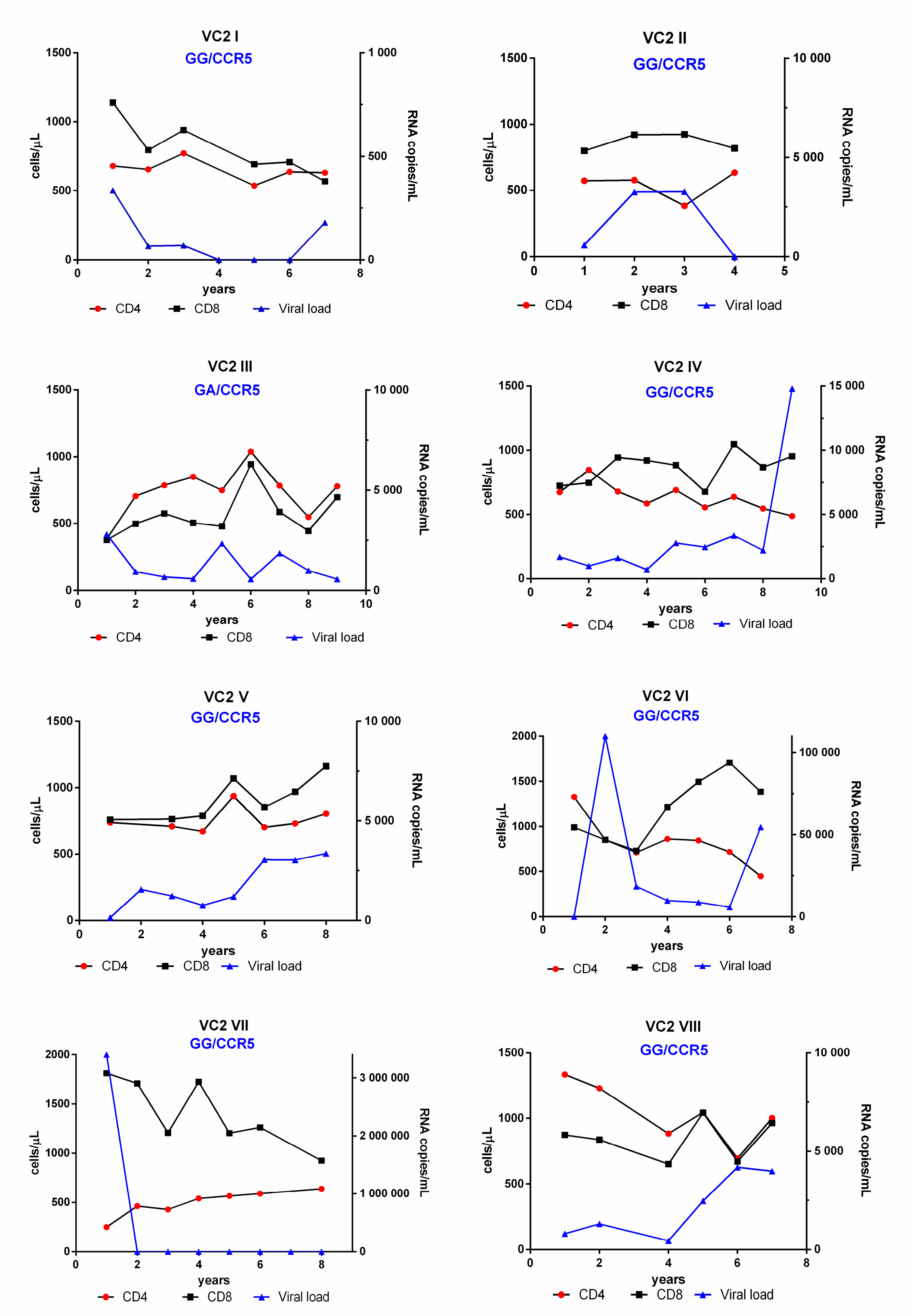

Supplementary Figure S2. Evolution of CD4⁺ TL and CD8⁺ TL quantification and HIV-1 plasma viral load from the year of diagnosis or the first available quantification among viremia controller 2 (VC2). GA: heterozygous for the *SDF1*-3′A variant. GG: wild homozygous for the *SDF1*-3′A.

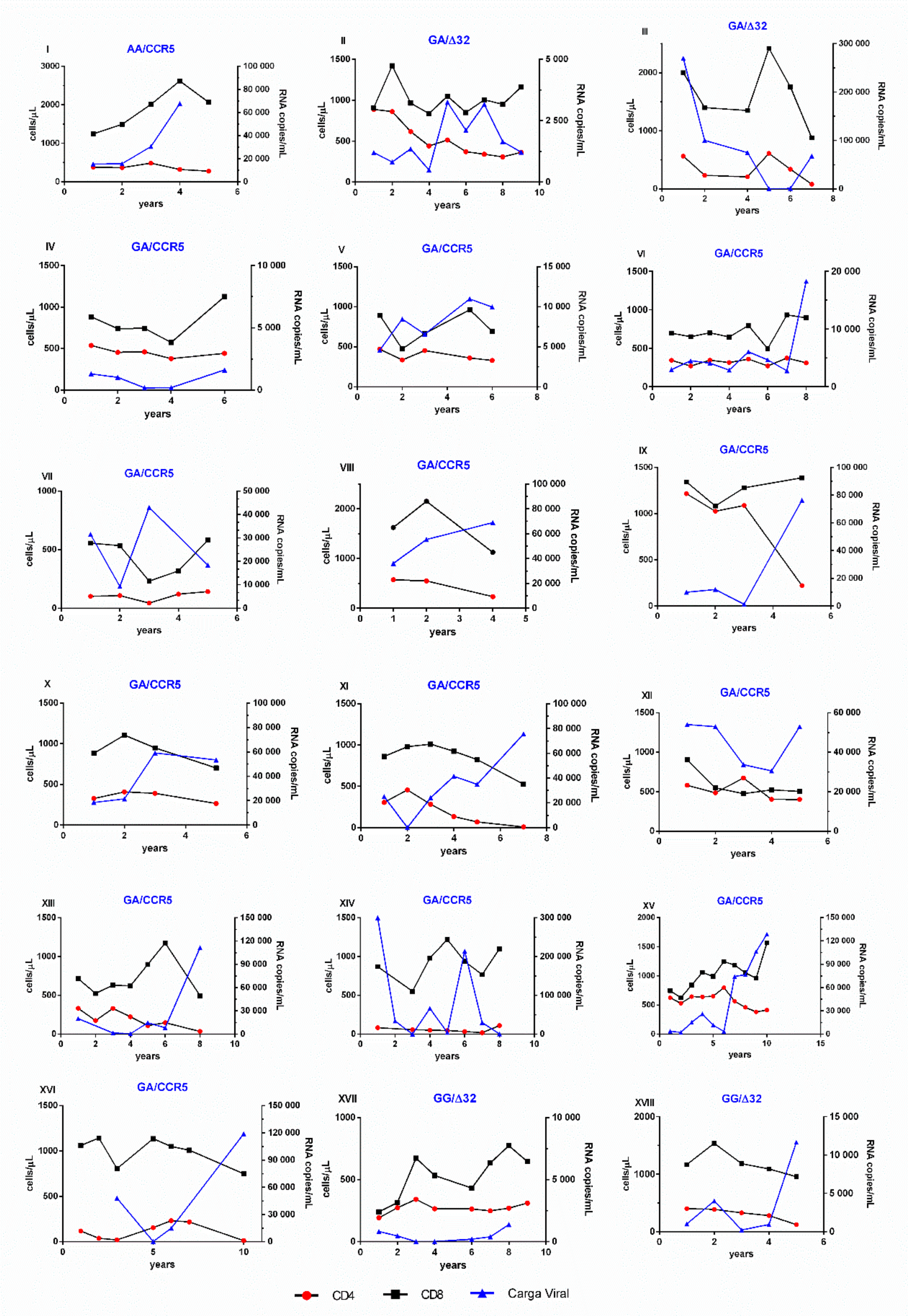

Supplementary Figure S3. Evolution of CD4+ TL and CD8+ TL quantification and HIV-1 plasma viral load, from the year of diagnosis or the first available quantification among viremia non-controllers (NC). AA: homozygous mutant for the *SDF1*-3′A variant. GA: heterozygous for the *SDF1*-3′A variant; Δ32: presence of the *CCR5*Δ32 variant.

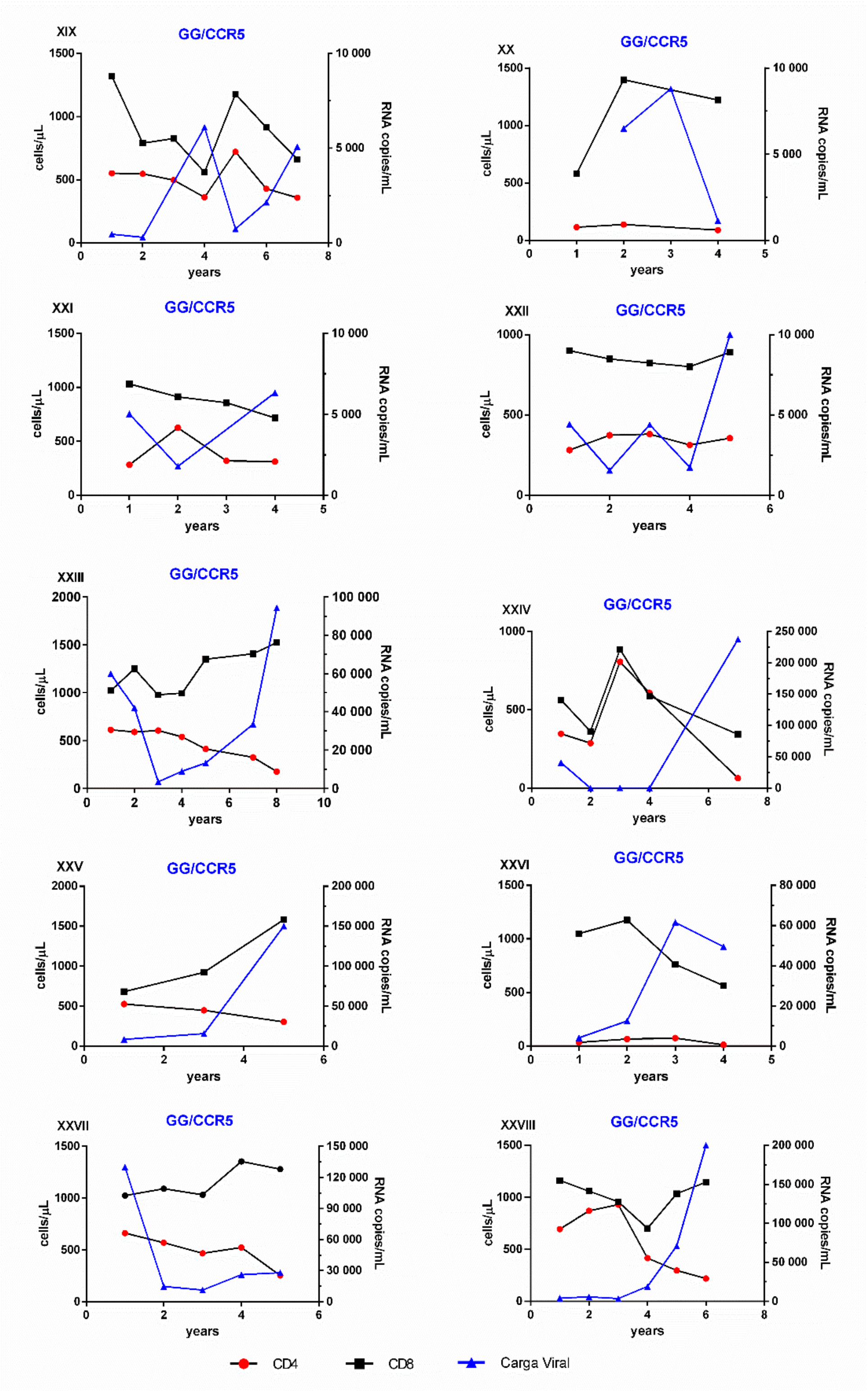

Supplementary Figure S3 (cont.). Evolution of CD4$^+$ TL and CD8$^+$ TL quantification and HIV-1 plasma viral load, from the year of diagnosis or the first available quantification among viremia non-controllers (NC). AA: homozygous mutant for the *SDF1-3'*A variant. GA: heterozygous for the *SDF1-3'*A variant; Δ32: presence of the *CCR5*Δ32 variant.
